# X-Ray Performance of SiC NPN Radiation Detector

**DOI:** 10.3390/mi16010002

**Published:** 2024-12-24

**Authors:** Jing Wang, Leidang Zhou, Liang Chen, Silong Zhang, Fangbao Wang, Tingting Fan, Zhuo Chen, Song Bai, Xiaoping Ouyang

**Affiliations:** 1 School of Microelectronics, Xidian University, Xi’an 710071, China; tracy1622@126.com; 2 Northwest Institute of Nuclear Technology, Xi’an 710024, Chinaoyxp2003@aliyun.com (X.O.); 3 School of Microelectronics, Xi’an Jiaotong University, Xi’an 710049, China; 4 Xi’an Engineering Research Center of Advanced 3D Vision, Biyuan 3rd Road, Xi’an 710000, China; 5 School of Materials Science and Engineering, Xiangtan University, Xiangtan 411105, China; 6 State Key Laboratory of Wide Bandgap Semiconductor Devices and Integrated Technology, Nanjing Electronic Devices Institute, Nanjing 210016, China

**Keywords:** radiation detection, semiconductor detector, bipolar transistor, gain

## Abstract

In this paper, a silicon carbide (SiC) phototransistor based on an open-base structure was fabricated and used as a radiation detector. In contrast to the exposed and thin sensitive region of traditional photo detectors, the sensitive region of the radiation detector was much thicker (30 μm), ensuring the high energy deposition of radiation particles. The response properties of the fabricated SiC npn radiation detector were characterized by high-energy X-ray illumination with a maximum X-ray photon energy of 30 keV. The SiC npn detector featured stable and clear response to the X-ray within 0.0766 Gy∙s^−1^ to 0.766 Gy∙s^−1^ below 300 V. Due to to the low leakage current of less than 1 nA and the fully depleted sensitive region, the bipolar-transistor-modeled SiC npn detector exhibited a clear common-emitter current gain of 5.85 at 200 V (under 0.383 Gy∙s^−1^), where the gain increased with bias voltage due to the Early effect and reached 7.55 at 300 V. In addition, the transient response of the SiC npn detector revealed a longer delay time than the SiC diode of the same size, which was associated with the larger effective capacitance of the npn structure. The npn detector with internal gain showed great potential in radiation detection.

## 1. Introduction

Semiconductor detectors have consistently played a vital role in the detection of X-ray and nuclear radiation [1,2,3]. In some cases, the signal produced by the interaction of rays with semiconductor detectors is often minimal, such as low energy ray detection, and energy spectra measurement, and may require amplification before being recorded. However, the embedded preamplifier can introduce additional noise, restricted signal bandwidth, and the loss of critical high-frequency and low-amplitude information, and it hardly improves the detection limitation of the system. It is essential to investigate detectors possessing internal gain to extend the energy detection limits effectively. This approach not only simplifies the system but also enhances the signal-to-noise ratio.

Avalanche diodes, photoconductors, and phototransistors are known for their internal gain properties and are extensively studied in photo detection [4,5,6]. For radiation detection, the random nature and long travel range of incoming rays and particles necessitate that the detector has a large, thick, and uniform sensitive region. This design is essential for effectively collecting the generated carriers. Additionally, the detector should maintain a stable and low leakage current to minimize noise interference. Consequently, the phototransistors show great potential in radiation detection. Although the silicon (Si)-based bipolar junction transistor (BJT)-structured radiation detectors have achieved a high internal gain of ~450 and a fast response below 50 μs [7,8,9,10], the applications of these detectors in radiation detection are still limited by the high leakage current density and low radiation tolerance of Si-based devices.

For decades, wide band gap semiconductors, owing to their low intrinsic carrier density and strong bonding energy, have provided low leakage current and high radiation resistance in devices and have drawn significant attention for the application of next-generation electron devices in space missions, military applications, and high-energy physics experiments [11,12,13]. Silicon carbide (SiC) is a promising candidate for next-generation commercial radiation detector applications due to its superior thermal conductivity, high chemical stabilities, mature material growth method, advanced fabrication technology, and low cost [14,15,16]. Recently, SiC-based electron devices have been widely used in consumer electronics, power electronics, and ultraviolet (UV) detection, and an increasing number of academic reports are focusing on SiC-based radiation detectors to explore their potential in the radiation environment [17,18,19].

In our previous report [20], a SiC-based npn radiation detector with an internal gain property was proposed. In this paper, more details of the response to X-ray illumination have been investigated, including the output current and response time under various bias voltages and dose rates, and the process of internal gain has been discussed.

## 2. Experiments

**Figure 1:**
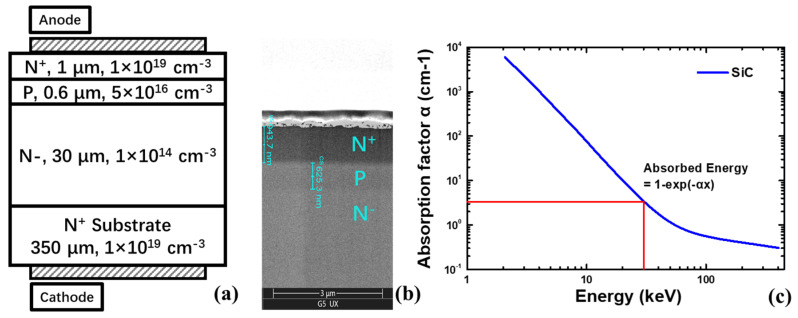
(**a**) Schematic of the SiC npn detector; (**b**) SEM image of the cross-sectional schematic of the npn structure; (**c**) absorption factor of SiC material.

The 4H-SiC npn detector was fabricated on a 6-inch n+ 4H-SiC substrate, where the epilayers, 1 × 10^14^ cm^−3^ nitrogen doping in 30 μm, 5 × 10^16^ cm^−3^ aluminum doping in 0.6 μm, and 1 × 10^19^ cm^−3^ nitrogen doping in 1 μm, were grown sequentially using the Metal Organic Chemical Vapor Deposition (MOCVD) method. After isolation by an Inductively Coupled Plasma (ICP) etching (>1 µm), ohmic contacts were formed on the top and bottom sides by using the evaporation of nickel and rapid thermal annealing (RTA) method. Figure 1a shows the schematic of the fabricated SiC npn detector. Finally, a passivation layer of 400 nmSiO_2_ was deposited using plasma-enhanced chemical vapor deposition (PECVD) on the top side, and the anode electrode was formed via holes etched on the SiO_2_ layer. Figure 1b shows the scanning electron microscope (SEM) image of the n+/p/n- structure, where the width of the fabricated n+ and p layers were extracted to be 943.7 nm and 625.3 nm, respectively.

The X-ray source (60 kV, 12W X-ray source, Moxtek, MAGPRO, Camden, TN, USA, Tungsten target) was used in the stable and switching X-ray measurements, and a B2902A (Keysight, Santa Rosa, CA, USA) provided by Xi’an Jiaotong University was used to record the output current of the detector. The bias voltage was applied on the cathode electrode in the experiment. To calibrate the new npn detector, a 4H-SiC p-i-n diode with an area of 1 cm^2^ and a fully depleted region of 30 μm at 80 V was used in the experiment. The p-i-n detector can achieve over 95% collection efficiency for photo-generated carriers in a fully depleted state and was used to calculate the internal gain of the SiC npn detector. More details can be found in our previous report [20]. The sensitive region of both the p-i-n and npn detectors was the depletion region of the reverse-biased p/n- junction. When the bias voltage increased to more than 80 V, the 30 μm n- layer was fully depleted.

**Figure 2:**
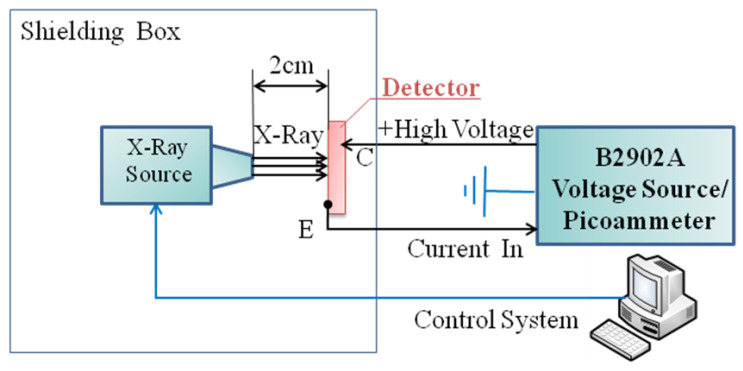
Schematic of the measurement system of responses to X-ray source.

Figure 2 shows the X-ray response experiment. When the X-ray tube accelerating voltage is set at 30 kV, the X-ray source can provide a wide continuous spectrum of X-ray beam with a peak photon energy at around 24 keV, a mean photon energy of 10 keV and a maximum photon energy of 30 keV. In this case, when the X-ray tube current is set at 100 µA, the X-ray dose rate is 0.383 Gy∙s^−1^ at a distance of 2 cm from the source, which was calibrated by a UNIDOS webline dosemeter. Furthermore, the X-ray dose rate is proportional to the X-ray tube current, which can be tuned from 1 to 400 µA. Figure 1c shows the linear attenuation coefficient of SiC material [21], which can also be also described as the absorption factor (α). Then, the absorbed X-ray energy (*E_SiC_*) of SiC material can be described by

(1) $$E_{SiC}=E_{0}\left(1-\exp\left(-\alpha x\right)\right)$$

where *E*_0_ is the total energy of the X-ray with certain photon energy, and *x* is the thickness of the SiC material.

The α of the SiC material was 69.1 cm^−1^, 5.7 cm^−1^, and 3.4 cm^−1^ for the X-ray photon energy of 10 keV, 24 keV and 30 keV, respectively. The stop range of the X-ray photon can be defined as the distance at which 95% energy was absorbed. Thus, the shortest stop range was calculated to be 434 μm, corresponding to a 10 keV photon energy, which is much longer than the 30 μm thickness of the sensitive region of the SiC npn detector. Thus, the difference in structure between the SiC p-i-n detector and the npn detector with the same sensitive region (30 μm) can be omitted.

## 3. Results and Discussion

**Figure 3:**
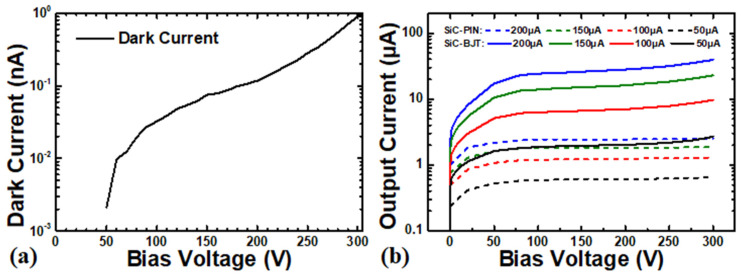
(**a**) Dark current of the fabricated SiC npn detector; (**b**) output current of the SiC npn detector and SiC pn diode detector at various tube currents [20].

Figure 3 shows the dark current-voltage (*I*-*V*) characteristic of the SiC npn detector and the output current of the SiC npn detector and SiC p-i-n detector at various tube currents (dose rate = 0.383 Gy∙s^−1^ when the tube current = 100 μA). The 1 cm^2^ detector exhibited a low leakage current < 1 nA at biased 300 V and higher outputs than the SiC p-i-n detector. To quantitatively evaluate the X-ray detection performances of the fabricated SiC npn detector, the sensitivity (*S*) and the noise-equivalent dose rate (*NED*) were further calculated. The sensitivity is defined as the ratio between the net response current (output current under X-ray illumination minus dark current, *I_x-ray_* − *I_dark_*) and the incident X-ray dose rate reaching to the detector (*D*) [22]:

(2) $$S=\frac{I_{X-ray}-I_{dark}}{D}$$

The noise-equivalent dose rate is defined as the incident X-ray dose rate that gives a signal-to-noise ratio of one in a 1-Hz bandwidth, which is a measure of the detection limitation of the detector and can be described by the following equation, where *q* is the unit charge:

(3) $$NED=D\frac{\sqrt{2qI_{dark}}}{I_{X-ray}-I_{dark}}$$

**Figure 4:**
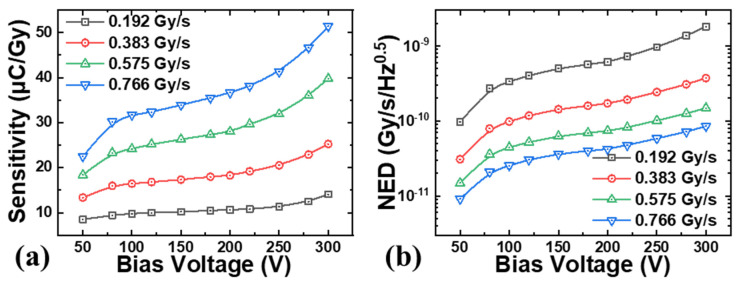
(**a**) The sensitivity (*S*) and (**b**) the noise equivalent dose rate (*NED*) of the SiC npn detector.

Figure 4 plots the sensitivity and noise-equivalent dose rate against the reverse bias voltage at various dose rates from 0.192 Gy∙s^−1^ to 0.766 Gy∙s^−1^ for the fabricated SiC npn detectors. The sensitivity increased with bias voltages, increasing more rapidly at higher biased voltages, and reached 51.41 μC∙Gy^−1^ at 300 V (36.7 μC∙Gy^−1^ @200 V) under 0.766 Gy∙s^−1^. Moreover, the sensitivity also increased with the dose rates at certain biased voltages. This phenomenon indicated a nonlinear output of the SiC npn detector against the dose rates, which limited the applications of dose monitor for the SiC npn detector. However, the noise-equivalent dose rate increased with the dose rates, showing a higher resolution of the X-ray signal at higher dose rates, which was attributed to the increasing sensitivity of the SiC npn detector. Besides, the detector featured a much lower noise-equivalent dose rate of 9.13 × 10^−12^ Gy∙s^−1^∙Hz^−0.5^ at 50 V under 0.766 Gy∙s^−1^. The noise-equivalent dose rate increased with the bias voltages because the dark current of the detector increased faster. On the contrary, the SiC p-i-n featured a linear output with respect to the dose rates [20], and the noise-equivalent dose rate increased with the bias voltages due to the increasing dark current but remained independent of the dose rates.

Notably, the output current of the SiC p-i-n detector was saturated beyond 80 V bias voltage, which was the fully depleted voltage of the 30 μm n- layer. Because there is no gain property for the p-i-n detector, the saturated output current of the SiC p-i-n detector can be attributed to the situation when the radiation-generated carriers were fully collected. In this case, the internal gain of the SiC npn detector, with the same sensitive region as the p-i-n detector, can be estimated by the following equation:

(4) $$Gain=\frac{I_{X-ray}-I_{dark}}{I_{pin,X-ray}-I_{pin,dark}}$$

where the *I_pin,X-ray_* is the output current and the *I_pin.dark_* is the dark current of the SiC p-i-n detector.

**Figure 5:**
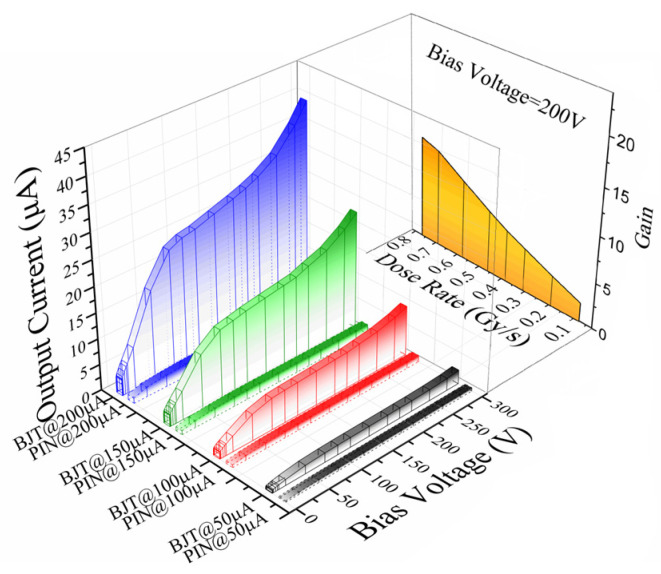
The internal gain properties against the dose rates at 200 V.

**Figure 6:**
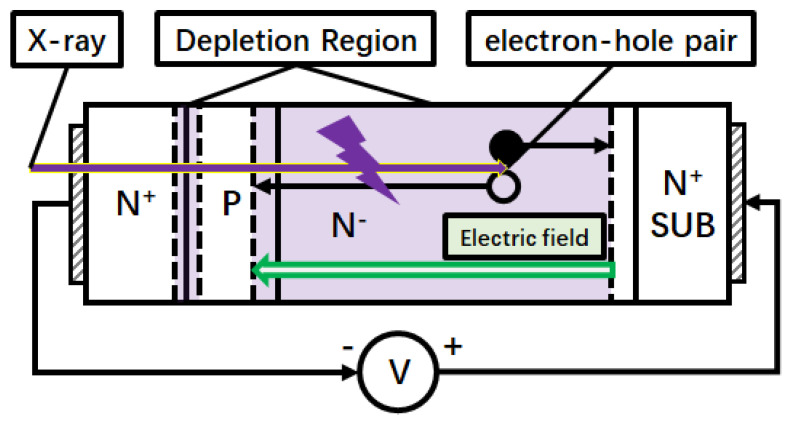
Schematic of the response process of the SiC npn detector under X-ray illumination.

Figure 5 shows the calculated internal gain of the SiC npn detector at 200 V. The internal gain of the detector increased with the bias voltages and the dose rates from 0.192 Gy∙s^−1^ to 0.766 Gy∙s^−1^. Figure 6 illustrates the carriers’ collection process and reveals the origin of the internal gain of the SiC npn detector. Because the concentration of the p region was three orders higher than that of the n- region, the depletion region was mainly applied on the n- side when the cathode electrode was biased. In this model, the X-ray radiation-generated carriers (electron-hole pairs) in the depletion region were collected, forming a response current towards the anode from the cathode. The collected holes converged in the neutral p region and then shifted up the potential of p region. This process generated a higher electron injection from n+ region, which diffused in the depletion region through the neutral p region. More carriers were collected than the X-ray generated across the electrodes, leading to the internal gain. This process was similar to the phototransistor [23], but the difference was the thicker depleted n- region requirement for radiation detection.

**Figure 7:**
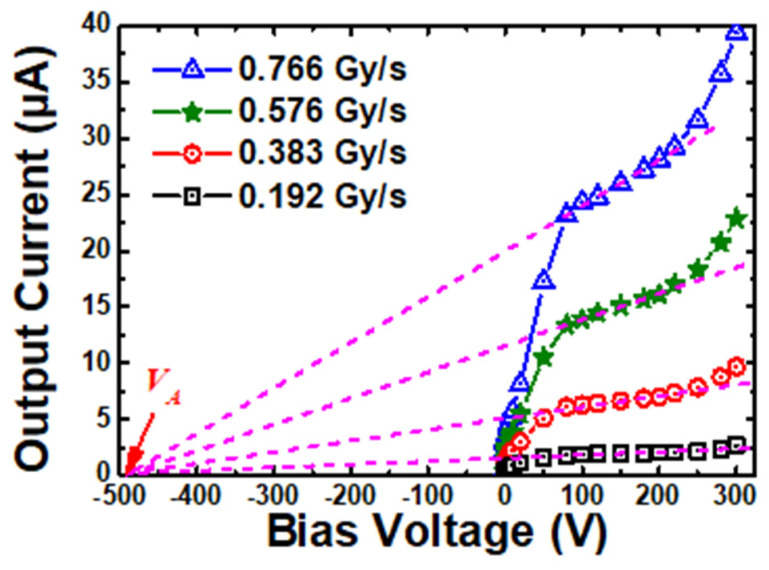
The extraction of Early voltage *V*_A_ of the SiC npn detector under X-ray illumination.

However, the design of the p region was much more challenging. On the one hand, the thicker depletion region required a high bias voltage and a low leakage current for radiation detectors to increase their charge collection efficiency. The biased voltages of the radiation detectors were much higher in many cases than the fully depleted voltage, and the width of p should be thick enough to sustain the high bias voltage. A thick neutral p region would eliminate the internal gain of the detector, but a thin one would be easy to punch through, leading to a large leakage current. On the other hand, the high concentration of the p region would solve the width problem but would also reduce the gain of the detector. Besides, the quality of the p region was also important because the internal gain was much more sensitive to the properties of the p region. In our study, the output current of the SiC npn detector was still increasing, whereas the output current of the SiC p-i-n detector was saturated beyond 80 V. This phenomenon was like the Early effect in BJT, which was caused by the insufficiently high concentration of the p region. A higher doping concentration of the layer will reduce this impact. Figure 7 shows the fitting curves of the increasing output currents across the bias voltages from 80 V to 200 V. All these fitting curves converged at one point (*V*_A_), which was extracted to be −490 V. In addition, the low concentration of the p region also resulted in higher leakage current and output current beyond 200 V, attributed to the thin neutral p region at high bias voltages. In short, both the width and concentration of the p region are crucial to the npn detector and will be further optimized in our future work.

**Figure 8:**
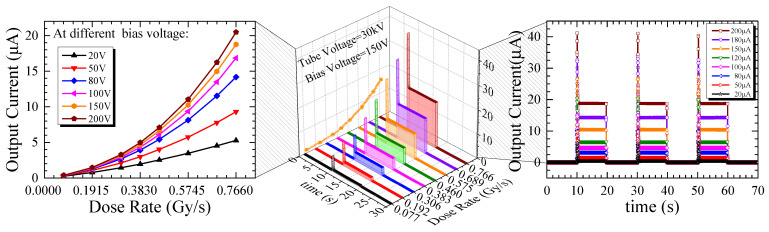
The output currents against time and dose rates of the SiC npn detector at 150 V; the inset on the left shows the output current against dose rates at various bias voltages, and the inset on the right shows the output current at various dose rates at 150 V respond to the switching X-ray illuminations.

**Figure 9:**
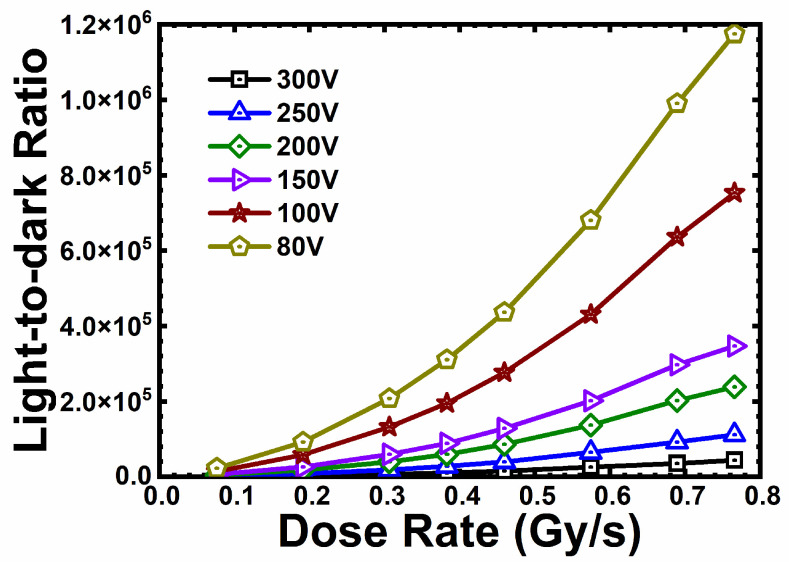
The light-to-dark ratio of the SiC npn detector against dose rates under various bias voltages.

Figure 8 shows the transient response of the SiC npn detector under X-ray illumination with a 20 s switching period. There were overshoots (with a rising edge of 10 ms) at the beginning of the transient output current curves of the SiC npn detector, which were generated by the X-ray source during the turned-on process [24].The SiC npn detector exhibited a repeatable and stable output waveform at 150 V in dose rates ranging from 0.077 Gy∙s^−1^ to 0.766 Gy∙s^−1^. The output current of the SiC npn detector was extracted when the response waveform was stable (Supplementary Materials, Figure S1). The left inset showed nonlinear outputs of the detector at dose rates from 0.077 Gy∙s^−1^ to 0.766 Gy∙s^−1^. The nonlinear properties were independent of the bias voltages. According to Figure 4a, the output current against dose rates exhibited a nonlinear property, which can be explained by the nonlinear transfer characteristic curve of BJT, as discussed above. This nonlinear property of the output currents versus the dose rates also resulted in the nonlinear increase of the light-to-dark ratio against the dose rates of the SiC npn detector, shown in Figure 9. The light-to-dark ratio of the SiC npn detector at 200 V and 0.766 Gy∙s^−1^ was calculated to be 238,643, much higher than that of the SiC p-i-n detector of 108. The high light-to-dark ratio of the SiC npn detector was attributed to the low leakage current and the internal gain. The ratio decreased with bias voltages from 80 V to 300 V because the leakage current increased faster when the n- region was fully depleted, especially beyond 200 V.

**Figure 10:**
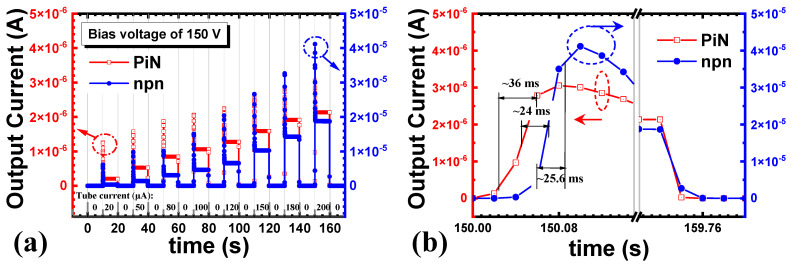
Output currents of SiC PiN and npn detectors biased at 150 V (**a**) at various dose rates under switching X-ray illuminations, (**b**) at the dose rate of 0.766 Gy∙s^−1^ in one period.

The comparison between the transient response of the SiC npn detector and p-i-n detector was made to investigate the response process of the SiC npn detector. Figure 10a shows the output currents of both detectors biased at 150 V. The sampling time was 20 ms. The leakage currents were comparable when both detectors were biased at 150 V. Both the output currents increased with the X-ray tube currents (dose rates), and the output current of the SiC npn detector approached 10 times that of the SiC p-i-n detector, gradually with the dose rates. Figure 10b shows one response and recovery process of both detectors at 0.766 Gy∙s^−1^ (Supplementary Materials, Figure S2). The transient response results showed that the response time and the recover time of the SiC npn detector was comparable to that of the SiC p-i-n detector. However, there was a delay in the response of the SiC npn detector, which might have been caused by the higher capacitance of the npn structure.

In order to broaden the applications of the npn detector, such as the dose rate monitor and communication, the nonlinear output characteristics should be emulated in the future. In the current npn structure, like an open-based BJT, the potential of the p layer was affected by the radiation-generated response current, leading to a floating gain of the detector. If the potential of the p layer can be clamped by involving a terminal on the p layer or by some other method, the linear output characteristics may be improved.

## 4. Conclusions

In summary, a vertical two-terminal SiC npn detector was fabricated using homoepitaxy SiC material. Due to the high quality of the SiC material and its 30 μm thick n- layer, the detector featured an internal gain based on its bipolar structure. The leakage current of the fabricated SiC npn detector was lower than 1 nA at 300 V. The detector exhibited a sensitivity of 51.41 μC∙Gy^−1^ at 300 V (36.7 μC∙Gy^−1^ @200 V) under 0.766 Gy∙s^−1^, a low noise-equivalent dose rate of 9.13 × 10^−12^ Gy∙s^−1^∙Hz^−0.5^ at 50 V under 0.766 Gy∙s^−1^ and a high light-to-dark ratio of 238,643 at 200 V and 0.766 Gy∙s^−1^. However, the internal gain showed dependence on the bias voltages and dose rates and involved nonlinear output currents against dose rates from 0.077 Gy∙s^−1^ to 0.766 Gy∙s^−1^. The design of the p layer was crucial to obtaining a stable gain and solving the trade off between the gain and the leakage current. Furthermore, the transient response of the SiC npn detector showed a fast response and recover time of around 20 ms and a delay of response due to the high capacitance of the npn structure. In short, the SiC npn has shown great potential in radiation detection with internal gain, but the internal gain process of the SiC npn detector sacrificed the linear output characteristics of the detector. More studies should be designed to further optimize the response properties in X-ray and radiation detection applications.

## Data Availability

The original contributions presented in the study are included in the article, further inquiries can be directed to the corresponding author.

## Supplementary Materials

The following supporting information can be downloaded at: https://www.mdpi.com/article/10.3390/mi16010002/s1, Figure S1: Output currents of SiC PiN biased at 150 V at various tube currents (dose rates) in five periods. Figure S2: Output currents of SiC PiN and npn detectors biased at 150 V at the dose rate of 0.766 Gy∙s^−1^ in one period.
