# Supplementary material for: X-Ray Performance of SiC NPN Radiation Detector
Source: Micromachines (Basel). 2024 Dec 24;16(1):2. doi: 10.3390/mi16010002 (PMC11767698; doi:10.3390/mi16010002)
Supplement: Supplementary file 1 [file micromachines-16-00002-s001.zip › micromachines-3359007-supplementary.pdf]

Supporting Information

# X-ray performance of SiC NPN Radiation Detector

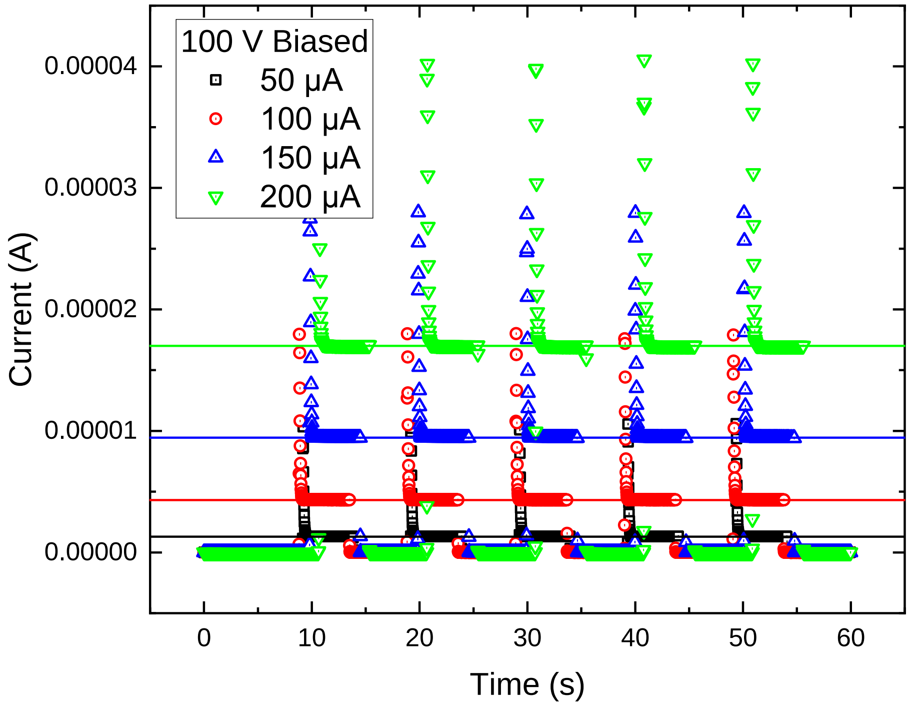


Figure S1 Output currents of SiC PiN biased at 150 V at various tube currents (dose rates) in five periods.

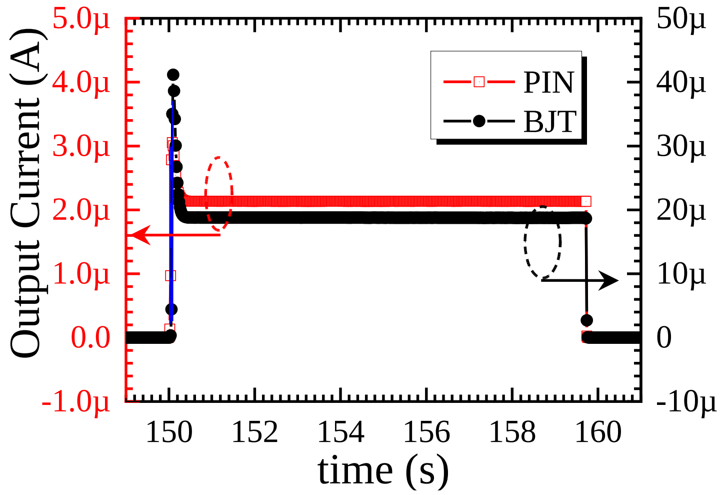


Figure S2 Output currents of SiC PiN and npn detectors biased at 150 V at the dose rate of 0.766 $Gy\cdot s^{-1}$ in one period.
